# Navigating Therapeutic Challenges in BRAF-Mutated NSCLC: Non-V600 Mutations, Immunotherapy, and Overcoming Resistance

**DOI:** 10.3390/ijms252312972

**Published:** 2024-12-03

**Authors:** Martina Bortolot, Sara Torresan, Elisa De Carlo, Elisa Bertoli, Brigida Stanzione, Alessandro Del Conte, Michele Spina, Alessandra Bearz

**Affiliations:** 1 Department of Medical Oncology, Centro di Riferimento Oncologico di Aviano (CRO), IRCCS, 33081 Aviano, Italy; 2 Department of Medicine (DME), University of Udine, 33100 Udine, Italy

**Keywords:** NSCLC, BRAF non-v600, immunotherapy, resistant mechanisms

## Abstract

Although rare in non-small cell lung cancer (NSCLC), BRAF mutations present considerable therapeutic challenges. While the use of BRAF and MEK inhibitor combinations has significantly improved survival outcomes in patients with BRAF V600E mutations, no targeted therapies are currently available for class II and III mutations, leaving the optimal treatment strategy and prognosis for these patients uncertain. Additionally, despite immunotherapy typically showing limited benefit in patients with other activating genomic alterations, it appears to deliver comparable efficacy in BRAF-mutated NSCLC, emerging as a potentially viable treatment option, particularly in patients with a history of smoking. However, resistance to BRAF pathway inhibitors is inevitable, leading to disease progression, and a well-defined strategy to overcome these resistance mechanisms is lacking. This review aims to explore the critical challenges in the management of BRAF-mutated NSCLC, providing a comprehensive summary of the current evidence and highlighting ongoing clinical trials that aim to address these critical gaps.

## 1. Introduction

In NSCLC, mutations of the V-Raf murine sarcoma viral oncogene homolog B (BRAF) gene are rare and detected in approximately 1.5–2.5% of patients [1]. These mutations are more frequently observed in never-smokers, women, and aggressive histological subtypes such as micropapillary adenocarcinoma [1].

**Figure 1:**
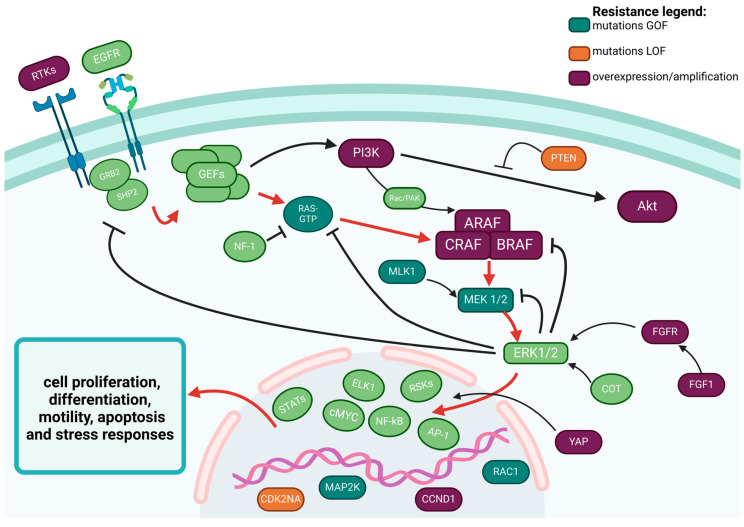
BRAF pathway with the most recognized resistance mechanism highlighted. Red arrows indicate the main signaling chain. **GOF**—*gain of function*; **LOF**—*loss of function*; **EGFR**—*Epidermal Growth Factor Receptor*; **RTKs**—*Receptor Tyrosine Kinases*; **GRB2**—*Growth Factor Receptor-Bound Protein 2*; **SHP**—*Src Homology Phosphatases*; **GEFs**—*Guanine nucleotide Exchange Factors*; **RAS**—*Rat Sarcoma*; **NF1**—*Neurofibromin 1*; **RAF**—*Rapidly Accelerated Fibrosarcoma*; **MEK**—*Mitogen-Activated Protein Kinase*; **ERK**—*Extracellular Signal-Regulated Kinase*; **PI3K**—*Phosphoinositide 3-Kinase*; **AKT**—*Protein Kinase B*; **PTEN**—*Phosphatase and Tensin Homolog*; **MLK1**—*Mixed-Lineage Kinase 1*; **FGF1**—*Fibroblast Growth Factor 1*; **FGFR**—*Fibroblast Growth Factor Receptor*; **COT**—*Cot/Tpl2*; **YAP**—*Yes-Associated Protein*; ***STATs***—*Signal Transducer and Activator of Transcription*; ***cMYC***—*Cellular MYC*; ***ELK1***—*ETS-Like Protein 1*; ***NF-kB***—*Nuclear Factor kappa B*; ***RSK***—*Ribosomal S6 Kinase*; ***AP-1***—*Activator Protein 1*; ***CDK2NA***—*Cyclin-Dependent Kinase Inhibitor 2A*; ***MAP2K***—*Mitogen-Activated Protein Kinase Kinase*; ***CCND1***—*Cyclin D1*; ***RAC1***—*Ras-Related C3 Botulinum Toxin Substrate 1*.

BRAF gene is located on chromosome 7 (7q34) and encodes a serine/threonine-protein kinase involved in the mitogen-activated protein kinase (MAPK) signaling pathway, which regulates cell growth and division. The pathway includes rat sarcoma (RAS)–rapidly accelerated fibrosarcoma (RAF)–mitogen-activated protein (MEK)–extracellular signal-regulated kinase (ERK) [2] (Figure 1). The signaling cascade begins when growth factors bind to receptor tyrosine kinases (RTKs), triggering RAS activation through GTP binding. Once activated, RAS activates RAF family kinases (ARAF, BRAF, and CRAF), which form functional homo- or heterodimers. These RAF kinases activate MEK, which subsequently phosphorylates and activates ERK, which influences various downstream targets and drives negative feedback mechanisms. Normally, the BRAF kinase is silenced via this negative feedback once the signal advances in the pathway; with BRAF mutations, the RAS–RAF–MEK–ERK pathway remains persistently active, driving uncontrolled cell growth and proliferation.

The most common BRAF mutations are reported in Table 1. With the increasing knowledge of the pathobiology of BRAF variants [2], three classes of BRAF mutations have been identified based on mutation site and function [3]. Class I mutations include nucleotide variants, which occur in the valine residue at amino acid position 600 of exon 15, most frequently p.V600E (point mutation T1799A). These mutations result in strong constitutive activation of the BRAF kinase and, therefore, of the MAPK pathway; the mutated BRAF kinase can signal as a monomer, and therefore, it is RAS-independent (RAS activation is even suppressed due to a negative feedback loop after ERK activation) [4]. Class II mutations, such as L597, G464, and K601 mutations, occur in the activation segment or P-loop; they constitute dimers characterized by high or intermediate kinase activity in vitro (ERK phosphorylation activity is lower than that seen in V600E mutations [5]), independently of RAS activation. Finally, class III mutations, including D594 and G466, are located in the P-loop, catalytic loop, or DFG motif; unlike the other two groups, they are associated with a lack of or impaired BRAF kinase activity. Thus, the activity of MAPK pathway signaling is dependent on heterodimerization with wild-type RAF protomers and is often enhanced via Raf-1 proto-oncogene CRAF activation. These variants rather serve as amplifiers of upstream signals depending on alternative RAS activation and occur in tumors with high RTK activity or NF1 loss-of-function mutations [6].

Although this classification is preclinically validated, its value for clinical prognostication is not well established. In routine clinical practice, BRAF mutations are usually classified as V600 mutations and non-V600 mutations.

Given the results obtained in patients with advanced melanoma [7,8], BRAF inhibitors, such as dabrafenib and vemurafenib, were also evaluated in patients with advanced NSCLC and BRAF mutations, showing promising outcomes [9,10,11]. However, due to the development of various resistance mechanisms and the associated skin toxicity with an enhanced rate of secondary skin malignancies, combination therapies have been explored, suggesting that dual blockade of the BRAF pathway using both BRAF and MEK inhibitors could result in a significantly greater efficacy. In a phase II trial enrolling patients (N = 57) with metastatic BRAF V600E–mutant NSCLC previously treated with chemotherapy, combination of dabrafenib 150 mg twice daily plus trametinib 2 mg once daily in continuous 21-day cycles provided an objective response rate (ORR) of 63.2%, including 2 (3.5%) patients with complete response (CR) and 34 (59.6%) with partial response (PR). The median progression-free survival (mPFS) rate was 9.7 months, and the 6-month overall survival (OS) rate was 82% [12]. Further better results were achieved in the untreated population (N = 36, ORR 64% with 6% CR and 58% PR, mPFS 14.6 months, 2-year OS rate 51%) [13]. Although no phase III trials comparing BRAF/MEK inhibitors and standard chemotherapy +/− immunotherapy are available, these data led to the approval of this combination as a first-line-strategy in patients with advanced NSCLC and BRAF mutation [14,15]. Recently, a combination of encorafenib (anti-BRAF) 450 mg once daily plus binimetinib (anti-MEK) 45 mg twice daily in 28-day cycles also showed clinically meaningful antitumor activity in two phase II trials, either in untreated (ENCOBRAF trial: ORR 66.7%, mPFS 11.1 months; PHAROS trial: ORR 75%, PFS 30.2 months) or pre-treated (PHAROS trial: ORR 46%, mPFS 9.3 months) patients with advanced BRAF-V600E NSCLC, and was approved in this setting [16].

However, both strategies are currently recommended and reimbursed only for patients with class I mutations. For those with non-V600 BRAF mutations, the benefit of targeted therapies remains debated and unclear, with chemotherapy still being the preferred treatment. Thus, the question remains open of whether a more targeted approach could also be identified for this subgroup.

Furthermore, immune checkpoint inhibitors (ICIs) now represent the standard of care (SoC)—as monotherapy or combined with chemotherapy—for patients with advanced NSCLC without activating genomic alterations (AGA). Although it has been generally recognized that ICIs would not provide significant benefits to patients with AGA, such as EGFR mutation [17], the efficacy in BRAF-mutated NSCLC remains to be evaluated.

Finally, as resistance to BRAF pathway inhibitors inevitably occurs, leading to disease progression, strategies to overcome resistance mechanisms due to activation of alternative pathways, with new drugs or new treatment combinations, are needed.

In this review, we discuss all these open issues and challenges that clinicians face daily in the treatment of patients with NSCLC and BRAF mutations, summarizing the current data and the ongoing clinical trials.

## 2. BRAF Non-V600E

### 2.1. Clinical and Molecular Features

Class II and III comprise half of BRAF mutations in NSCLC, and the most frequent are the G469 group and K601E [18]. Compared to V600E mutations, they are most likely observed in non-smokers [19,20] and are associated with a higher risk of developing brain metastases (*p* ≤ 0.01) [21]. 

Different retrospective analyses reported no difference in OS between BRAF V600 and non-V600 mutations [20,22], although shorter OS is sometimes described in patients with class III mutations [23]. 

As per molecular profile, concurrent mutations are more frequently detected with BRAF class II and III mutations, especially class III. The most frequent co-mutations occur in TP53, EGFR, KRAS, and NF1 [3,18,22]. Tumor molecular burden (TMB) appears to be higher in BRAF non-V600E mutant NSCLC, though programmed death-ligand 1 (PDL-1) expression is more often <50% [19]. However, this finding is not consistent across different studies [3]. 

In terms of treatment responses, no significant differences have been observed in the effectiveness of chemotherapy across the various mutation classes [21,24]. However, in the prospective lung cancer genomic screening project (LC-SCRUM-Asia) enrolling 380 BRAF-mutated NSCLC (119 class I, 122 class II and 86 class III), PFS in response to platinum-containing chemotherapies (5.3 vs. 11.5 months, *p* < 0.01) and OS (14.5 vs. 34.8 months, *p* < 0.02) was significantly shorter in patients with class III as compared with class I [25].

### 2.2. Treatment Strategies

Registrative trials for dabrafenib plus trametinib and encorafenib plus binimetinib in NSCLC enrolled exclusively patients with BRAF V600E mutations [12,26]. Most data about the treatment of patients with BRAF non-V600 mutations derive from clinical case reports, a summary of which is reported in Table 2. Clinical trials and meta-analyses are difficult to interpret due to the wide range of mutations, drugs, and patient subgroups considered.

In the phase II BEAVER trial, 23 patients (2 NSCLC) with advanced solid tumors harboring non-V600E mutations, not previously treated with targeted therapy, received encorafenib and binimetinib. ORR was 13% (3/23), and mPFS was 2.4 months. BRAF mutation class was not associated with differences in PFS or ORR, while co-mutation in TP53 was associated with shorter PFS (1.8 vs. 4.0 months, *p* = 0.008) [40].

In another single prospective trial, 118 patients with BRAF-mutated NSCLC, of which 17 BRAF non-V600E (4 G466, 4 G469, 1 G596, 5 K601, and 3 N581), were treated with vemurafenib. Only one patient achieved stable disease, with a global ORR of 5.9% and a mPFS of 1.8 months [41].

A large meta-analysis examined 238 (58 NSCLC) patients with class II or class III BRAF mutations who received MAPK-targeted therapy. The ORR differed between BRAF mutation classes (41% vs. 13% in class II and class III, respectively; odds ratio 5.12, *p* = 0.002; in NSCLC patients, *p* = 0.018). In addition, mPFS was longer in class II mutated cases (4.6 vs. 2.1 months, HR 0.537, *p* = 0.001; in NSCLC patients, *p* = 0.028). Class II mutant patients also had a better response both to the combination of BRAF and MEK inhibitors or to MEK inhibitors alone compared to class III mutations (RR 56% vs. 27% and 56% vs. 9%, respectively) [29]. The combination of BRAF and MEK inhibitors was associated with the longest mPFS (5 months) in the whole study population [42].

In a retrospective multicohort study that included 35 patients treated with anti-BRAF monotherapy, 6 had BRAF non-V600E mutations (G466V, G469A, G469L, G596V, V600K, and K601E) [30]. Except for one patient with G596V who achieved PR with vemurafenib, all patients with non-V600E mutations (all located outside of the BRAF kinase domain’s activation segment) were resistant to anti-BRAF treatment [10]. Finally, in a retrospective analysis of 30 patients with BRAF nonV600E mutated NSCLC, G469A and L597R mutations (that are associated with high kinase activity) were associated with response to BRAF + MEK inhibitors. Median PFS for patients who received targeted therapy was 3.6 months (95% CI, 1.5–6.7), and mOS was 7.1 months (95% CI, 1.8-NR) [43]. The efficacy of dabrafenib and trametinib in those harboring G469A and G466V mutations was confirmed in NSCLC cellular lines [44].

### 2.3. Future Perspectives/New Strategies

Dedicated trials and, more importantly, a differentiated approach for the various BRAF mutations—based on a deeper understanding of how they alter intracellular pathways—are necessary to ensure truly targeted therapies. Current research is increasingly moving in this direction.

A phase I/II study of binimetinib and encorafenib in advanced solid tumors with BRAF non-V600E activating mutations has recently completed its accrual, and results are awaited (NCT03843775).

New generations of pan-RAF inhibitors (NCT02974725) and novel RAF-selective TKIs (NCT02428712, NCT05538130) are currently under-evaluation. Exarafenib, for example, is a highly selective pan-RAF inhibitor that demonstrated an encouraging response rate of 64.7% in a phase I clinical trial that is currently ongoing; the same trial is also evaluating the combination of exarafenib and binimetinib [45].

As stated, class II BRAF mutations form constitutively active RAF dimers. Therefore, the inhibition of the dimer rather than the BRAF monomer could be effective. Lifirafenib is a BRAF dimer inhibitor that showed promising results in combination with the novel MEK inhibitor mirdametinib, with a synergistic effect. In a dose-finding cohort comprising three patients with BRAF non-V600E mutations (but none of them with NSCLC), the combination showed an ORR of 27.8% [46]. BGB-3245 is another dimer inhibitor; in a phase Ia/b study enrolling patients with various solid tumors harboring BRAF class II mutations, it showed a DCR of 79% and an ORR of 18% in the overall population [47]. Further investigation of this drug is ongoing (NCT05580770), as well as other selective pan-RAF dimer inhibitors (belvarafenib [NCT03284502], naporafenib [NCT02607813]). A combination of trametinib with lifirafenib and naporafenib suppressed cell growth in the L597V and G469A cell lines more effectively than combinations of cobimetinib plus vemurafenib and binimetinib plus encorafenib [18] supporting the emerging role of these molecules. Preliminary results of PF-07799933—another BRAF dimer inhibitor—in BRAF non-V600E mutated patients are negative (8 patients, with no response) [48].

As class III BRAF mutations rely on RAS activity, which in turn depends on feedback from ERK 1/2, targeting ERK presents another potential therapeutic approach currently under investigation. In patients with BRAF nonV600 mutations, ulixertinib—a reversible, ATP-competitive ERK1/2 inhibitor with high potency and ERK1/2 selectivity—demonstrated favorable activity in early studies [49]. In a phase II study (NCT04488003), however, mPFS resulted in 1.5 (1.0–2.5) months and mOS 5.2 (3.8–7.0), leading to a premature discontinuation of the trial. Nonetheless, an expanded access program (EAP) for patients with solid tumors and a mutation in the MAPK pathway remains available (NCT04566393), and other ERK1/2 inhibitors are being evaluated (NCT02857270). Unfortunately, a limited antitumor activity, despite the observed on-target pharmacodynamic effect, was recently noted in a phase Ib trial regarding the combination of rineterkib—another ERK1/2 inhibitor—and naporafenib (n = 101, 3 PR) [50].

Other strategies include targeting ULK 1/2, a kinase involved in the autophagy initiation signaling that is inhibited by the inhibition of the MAPK pathway [51]. Phase I-II studies are underway to explore this option (NCT04892017).

A specific cohort for BRAF class II or III mutations is included in a phase I study (NCT05538130) evaluating PF-07799544, a next-generation MEK inhibitor alone or in combination with other agents in patients with solid tumors [52]. Targeting Shock Heat Protein 2 (SHP2) phosphatase with RMC-4550, a small-molecule allosteric inhibitor, resulted effective in human cancer models with class III BRAF mutations [53].

## 3. Role of ICIs in Treatment of BRAF Mutated NSCLC: Is This an Option?

### 3.1. Rationale

The efficacy of ICIs is well established in BRAF-mutant melanoma, but data regarding NSCLC are uncertain and are mainly derived from retrospective studies and case series.

In the subgroup of patients with BRAF mutation (n = 43) included in the IMMUNOTARGET registry, ORR was 24% and mPFS 3.1 months, supporting the possible efficacy of ICIs in this subgroup [54]. No significant difference between BRAF V600E and non-V600E mutations in terms of mPFS was observed, while previous or current smokers had better PFS (4.1 vs. 1.9 months) compared to ones who had never smoked [54]. Similarly, the retrospective chart review by Dudnik et al. analyzed 39 patients with BRAF-mutant NSCLC (21 with V600E mutation and 18 with non-V600E), reporting an ORR of 25% and 33% and a mPFS of 3.7 months and 4.1 months, respectively, in who received ICIs in the two groups [55]. BRAF-mutational status was not related to the probability of response to immunotherapy or PFS [55]. Of note, a positive correlation was observed between ICI exposure and OS: mOS was not reached in those treated with nivolumab, pembrolizumab, or atezolizumab monotherapy, and was 21.1 months in those who did not receive ICIs (*p* = 0.018) [55]. However, imbalances between the ICIs-treated and ICIs-untreated groups (higher PD-L1 expression and TMB in those treated with ICIs, number of previous therapy, and performance status) limit the value of the observation. A sub-analysis of patients (n = 11) with BRAF-mutant NSCLC enrolled in the Italian EAP of second-line nivolumab supported a comparable OS benefit with ICI treatment, regardless of BRAF mutational status (11.2 months in the BRAF wild-type subgroup and 10.3 months in BRAF mutated) [56].

Other studies confirmed the possible efficacy of immunotherapy in patients with NSCLC and BRAF mutations but suggested that it is limited to those with non-V600 mutations. Although a similar OS was noticed for patients treated with ICIs both in the BRAF-mutants and in the BRAF wild-type group (10 vs. 11 months, respectively, *p* = 0.334), subgroup analyses of the study by Zhang et al. revealed that the median survival was 14 months in BRAF non-V600E patients and 5 months in the V600E group (*p* = 0.017) [57]. Also, another retrospective study highlighted lower ORR and mOS (26% vs. 35% and 12.5 vs. 22 months, respectively) in the V600E cohort (n = 26) compared to the non-V600 one (n = 18) in patients with BRAF-mutated NSCLC receiving anti-PD-1 monotherapy [58]. More recently, a retrospective analysis of NSCLC patients receiving ICI as first-line showed a mPFS and mOS of 7.7 and 27.3 months, respectively, in those with BRAF non-V600E mutations (n = 10), notably longer than the outcomes achieved in the BRAF V600E population (n = 5, mPFS 3.9 months, ORR 40%) [59]. A profound and sustained response has been documented in a patient with NSCLC and BRAF G469A mutation receiving second-line nivolumab [34]. Although several other studies explored the outcomes of different classes of BRAF mutations [16,60] treated with ICIs, evidence remains inconsistent, and at present, it is not possible to identify subgroups of patients more likely to benefit from this approach.

Even though there are no prospective trials to confirm it, all the data reported above suggest that immunotherapy has efficacy in BRAF-mutant NSCLC patients and is not different from that observed in the overall NSCLC population. The possible benefit of ICI-based treatments has recently been evidenced not only in the advanced setting but also in the early stage. In a recent analysis, BRAF mutations (both V600 and non-V600) were identified in 4 (3.45%) plasma and tissue samples from 116 resectable stage IIIA/B NSCLC patients included in NADIM and NADIM II clinical trials, all of which were cases treated with neoadjuvant chemoimmunotherapy. Although mPFS and mOS were not reached for either BRAF-wild type or BRAF-mutated NSCLC, PFS and OS probabilities at 36 months were 60.5% and 76.1% for patients with BRAF-wild type tumors, while all those with BRAF mutation had no evidence of disease and were alive at data cut-off [61]. Likewise, BRAF mutations were significantly associated with a higher rate of pathological CR (pCR) after neoadjuvant treatment with chemo-ICIs (100% vs. 44.3%, RR: 2.26; 95% CI 1.78–2.85; *p* < 0.001) [61]. Achieving pCR after neoadjuvant chemo-ICIs is strongly associated with prolonged survival [62]. However, the small sample size does not allow for the establishment of definitive conclusions.

The better outcomes with ICIs in BRAF-mutated NSCLCs compared to other NSCLCs with AGA are probably due to a higher proportion of current or former smokers [63]. Smoking status should be considered in the evaluation of an ICI-based treatment as an increased efficacy of immunotherapy was evidenced in smokers, even in patients with oncogenic driver mutations [64]. Smoking-related cancers often harbor a ‘hot’ immune microenvironment, with higher PD-L1 expression and TMB. PD-L1 expression has a recognized predictive value for response to ICIs in NSCLC [65], and high TMB has emerged as a predictive biomarker for immunotherapy in multiple solid tumors [66]. High PD-L1 expression (>50%) was observed in up to 50% of patients analyzed (n = 29) in the Dudnik et al. trial (42% in the V600E cohort and 50% in the non-V600E); 2/11 patients showed high TMB [55]. Zhang et al. reported higher levels of TMB in BRAF-mutated NSCLC compared to the wild-type population (*p* = 0.009) but no significant differences in PD-L1 expression (*p* = 0.198) [57]. Although a significant association between PD-L1 expression and BRAF mutational status was not demonstrated by Provencio et al., a trend was observed; 2 of 3 BRAF-positive tumors also showed a TMB ≥ 20 [62]. More recently, 33.3% of the BRAF-mutated patients (n = 29) analyzed in the Italian ATLAS audit presented a PD-L1 expression rate > 50% [67].

In conclusion, based on the current evidence, immunotherapy should be considered for patients with NSCLC and BRAF mutations.

### 3.2. When to Consider ICIs

An increasingly important question is how to integrate ICIs and targeted agents into a potential therapeutic algorithm, particularly given early data suggesting that the combination of immunotherapy and TKIs in NSCLC may raise significant safety concerns [68].

While current guidelines recommend the combination of anti-BRAF and anti-MEK therapy as first-line treatment for patients with V600 mutations, single-agent ICIs or combined with chemotherapy (depending on PD-L1 status) should be used as first-line treatment for non-V600 patients. In patients with advanced BRAF V600E NSCLC, combinations of dabrafenib–trametinib or encorafenib–binimetinib should be offered upfront, as they are the only regimens with satisfying prospective data in this molecular subset of patients; immunotherapy should be a reasonable option after failure of target therapy. Single-agent ICIs with optional chemotherapy could be considered in patients with a smoking history, while chemotherapy with optional immunotherapy might be suggested for those without a smoking history. As for metastatic NSCLC without AGA, PD-L1 expression can also guide the decision. Moreover, immunotherapy may be proposed as first-line treatment in those countries where targeted therapies are not reimbursed in this setting or in specific cases unfit for TKIs and with features suggesting good outcomes achievable with ICIs (i.e., high PD-L1 or TMB, smoking history).

Starting with BRAF + MEK inhibition may reduce tumor burden and induce a tumor “priming” with the host, paving the way for a subsequent immunotherapeutic approach. Indeed, preclinical data have demonstrated that therapy targeting the MAPK pathway may alter and induce a more favorable tumor microenvironment. MEK inhibitors can upregulate major histocompatibility complex (MHC) I expression and enhance CD8+ T-cell infiltration into tumors (capable of recognizing and destroying tumor cells) [69]. Additionally, BRAF inhibition is associated with an increased CD4+ and CD8+ lymphocyte infiltrate, higher levels of granzyme B and perforin (which augment cytotoxicity), reduced release of immunosuppressive cytokines such as interleukin-6 and interleukin-8, and decreased levels of myeloid-derived suppressor cells [70]. All these immunological changes may boost the tumor responses driven by checkpoint blockade, supporting a synergistic activity of BRAF/MEK inhibitors and anti-PD(L)-1 agents. The combination of avutometinib—a unique RAF/MEK inhibitor that strongly inhibits MEK kinase activity and forms a dominant-negative RAF/MEK complex, preventing MEK phosphorylation by ARAF, BRAF, and CRAF—and an anti-PD-1 antibody demonstrated enhanced antitumor efficacy and prolonged survival in mouse models compared to the two strategies administered separately [71].

Given the promising results achieved in melanoma [72], ongoing trials are evaluating the combination of immunotherapy and TKIs in patients with NSCLC and BRAF V600 mutation.

In the subgroup with NSCLC (n = 28) of a phase Ib study investigating the combination of atezolizumab plus cobimetinib, a mOS of 13.2 months and an ORR of 18% were noted, irrespective of BRAF mutational status (only five patients had BRAF mutated NSCLC) [73]. After these results, cohort E of the phase II/III B-FAST trial (NCT03178552) aims to assess the efficacy (primarily in terms of time to response) and safety of the combination of atezolizumab, vemurafenib, and cobimetinib in patients with advanced NSCLC and BRAF V600 mutations. In the same subgroup, the phase Ib/II umbrella study Landscape 1011 (NCT04585815) will evaluate the safety and the antitumor activity of subcutaneous sasanlimab (a PD-1 antagonist monoclonal antibody) combined with encorafenib and binimetinib. Phase I is still ongoing, and phase II will focus only on previously untreated patients.

## 4. Overcoming BRAF Resistance in NSCLC

### 4.1. Resistance Mechanism

Despite the clinical success of BRAF and MEK inhibitors, clinical trials and real-world data demonstrate that their efficacy is limited by the development of adaptive and acquired resistance within one year. Data on mechanisms of resistance to BRAF/MEK inhibition are scarce in NSCLC and primarily derived from previous studies conducted in BRAF-mutated melanoma.

The major cause of adaptive drug resistance is the restoration of the MAPK pathway and, therefore, continuous reactivation of ERK signaling. Receptor tyrosine kinases (RTKs) upregulation, secondary mutations of NRAS and KRAS, BRAF amplifications (through either extrachromosomal DNA or intra-chromosomal homogeneously staining regions), or mutations with splice variants, C-RAF or A-RAF overexpression and MEK1/2 mutations have been previously described in melanoma as possible alterations responsible for this MAPK signaling restoration [74,75].

Acquired mutations in KRAS represent the most common off-target resistance mechanism. A case report of a patient with BRAF V600E-mutated advanced NSCLC treated with dabrafenib monotherapy who progressed after an initial response to BRAF inhibition reported a newly acquired KRAS G12D mutation [76]. In another case report, a KRAS c.35G>T p.(G12V) mutation was detected in a rebiopsy specimen of a patient after progression to dabrafenib plus trametinib treatment; of note, a retrospective analysis of KRAS status in the original biopsy specimen demonstrated that a KRAS mutation was already present in a minute subclone (0.26% fractional abundance) at time of diagnosis [77]. An NRAS Q61K mutation was identified in both tissue and plasma of another patient with NSCLC who progressed on dual BRAF/MEK inhibition [78]. More recently, complete molecular profiling conducted at resistance to BRAF/MEK inhibitors in 7 patients from the MATCH-R study identified MEK K57N, KRAS Q61R, and NRAS Q61K mutations [79].

The bypass activation of other associated signaling pathways is considered another mechanism of adaptive resistance. A partial crosstalk exists between the MAPK pathway and the phosphatidylinositol 3-kinase (PI3K)/protein kinase B (AKT) pathway; RTKs overexpression, phosphatase and tensin homolog (PTEN) loss of function, or activating mutations in phosphatidylinositol 3-kinase catalytic subunit (PI3KC) and AKT have been found to be responsible for resistance to BRAF inhibition [80,81].

Additionally, in a preclinical study of BRAF-mutant cell lines resistant to dual BRAF/MEK inhibition, an adaptive resistance mechanism mediated through the fibroblast growth factor receptor (FGFR) was noted. Two of the four patients analyzed demonstrated an increase in FGF1 expression at progression, resulting in autocrine activation of FGFR, which potentiates ERK activation [82]. FGFR overexpression has also been reported as a resistant mechanism to MEK inhibition in KRAS-mutated NSCLC models [83]. The Hippo pathway effector YAP (encoded by YAP1) was identified through a genetic screen in BRAF-mutant tumor cells as an alternative survival mechanism contributing to resistance to anti-RAF and anti-MEK therapy [84]; increased expression of YAP was a biomarker of worse initial response to RAF and MEK inhibition in patients in tumors harboring BRAF V600E mutations [84].

More recently, elevated expression of cyclin-dependent kinase 4 (CDK4) has been reported in patients with NSCLC progressed after dabrafenib-trametinib therapy [85]; however, the precise mechanisms underlying resistance acquisition have yet to be clarified. Another case report revealed the appearance of MET exon 14 skipping mutation and MET amplification in a patient with oligoprogression during BRAF and MEK inhibition [86], suggesting the possible role of MET alterations in the acquired resistance to these drugs.

Other mutations in mixed lineage kinase 1 (MLK1), Ras-related C3 botulinum toxin substrate 1 (RAC1), mitogen-activated protein kinase kinases 1 and 2 (MAP2K1 and MAP2K2, genes that encode for MEK1 and MEK2), amplification of CCND1 and loss of function of CDKN2A also contribute to resistance to RAF and MEK inhibitors [87,88].

The tumor microenvironment could also play a role in the development of resistance. Stromal cell secretion of hepatocyte growth factor (HGF) resulted in activation of the HGF receptor MET, reactivation of the MAPK and PI(3)K–AKT signaling pathways, and immediate resistance to RAF inhibition [89]. Similarly, macrophage-derived TNFα provides resistance to MAPK pathway inhibitors through overexpression of the microphthalmia transcription factor (MITF) [90]. The production of vascular endothelial growth factor (VEGF) may also lead to ERK pathway reactivation, bypassing BRAF inhibition [91].

Finally, other potential targets emerging on BRAF/MEK-resistance were reported in melanoma patients, which may open further treatment options. Three patients initially progressed on immunotherapy as well as BRAF/MEK inhibitors exhibited partial to near-complete responses to poly (ADP-Ribose) polymerase inhibitor (PARPi) therapy combined with BRAF/MEK inhibitors, highlighting the potential synergistic efficacy of this approach [92].

### 4.2. New Strategies

Several approaches to prevent the development of resistance and/or provide therapeutic alternatives to patients progressed after BRAF and MEK inhibitors are under evaluation (Table 3).

As previously mentioned, ~70% of mechanisms of resistance involve reactivation of the MAPK pathway, resulting in activation of the final kinase, ERK. Downstream inhibition of ERK represents a promising approach to suppress any upstream pathway activation or reactivation. A combination of RAF, MEK, and ERK inhibitors effectively suppressed tumor growth in xenograft models derived from samples of both targeted therapy-naive and pre-treated patients with melanoma or lung cancer and BRAF V600E mutations [93]. LY3214996, a potent, selective, ATP-competitive ERK1/2 inhibitor, demonstrated antitumor activity in BRAF-mutant models with acquired resistance in vitro and in vivo [94]. More recently, in a phase II basket trial combining LY3214996 and the CDK 4/6 inhibitor abemaciclib, stable disease was achieved in 33% of patients, with a median disease control rate of 6.5 months. Six of the 12 patients enrolled harbored pathogenic alterations in BRAF, but only 1 had NSCLC [95]. Although some other studies on the combination of BRAF or MEK inhibitors and CDK 4/6 inhibitors demonstrated promising antitumor activity in melanoma, this was accompanied by significant toxicity, limiting the applicability of this strategy [96].

Multi-inhibition of the other different intercommunicating pathways was also investigated in order to delay the acquisition of resistance. Given the crosstalk between their intracellular routes, therapeutic blockade of the PI3K/AKT/mTOR pathway along with the MAPK pathway is a promising treatment approach. The combination of vemurafenib and everolimus (anti-mTOR) demonstrated promising activity in 20 patients with BRAF-mutant advanced cancers (1 NSCLC), including those previously treated with a BRAF inhibitor [97]. Avutometinib is under evaluation combined with everolimus in RAS-RAF-MEK pathway mutant solid tumors (NCT02407509). The combination of RAF or MEK inhibition with FGFR inhibitors [82] or YAP inhibition [84] appears promising in preclinical investigations, too.

SHP2 phosphatase is a signal transduction node downstream of multiple RTKs and regulates RAS and MAPK signaling. PF-07284892, an allosteric SHP2 inhibitor, combined with encorafenib and binimetinib, overcame bypass-signaling-mediated resistance in various tumor models, including BRAF V600E-mutant colorectal cancer, presenting a valuable model for testing novel drug combinations [98].

Next to new combinatorial strategies, new small molecule inhibitors and targeting agents may help prevent or delay the development of therapeutic resistance. Paradox breakers are novel BRAF V600E inhibitors designed to inhibit BRAF mutant cells impairing BRAF homo/hetero-dimerization but to evade the paradoxical MAPK activation [99]. The next-generation type 1.5 (αC-helix OUT) RAF inhibitor PLX8394 showed efficacy in treatment-naive BRAF-mutated NSCLC and in cases with acquired vemurafenib resistance, in both V600E and certain non-V600 models [100]. Currently, HLX208, a novel RAF monomer-selective inhibitor, is being evaluated in combination with trametinib in solid tumors (NCT04965220). A combination of RAF dimer inhibitor lifirafenib with MEK inhibitor mirdametinib showed antitumor activity in pre-treated several solid tumor types harboring BRAF mutations, including one patient with BRAF V600E NSCLC [46]. Pan-RAF inhibitors can inhibit both active dimers and active monomers with similar potency, along with other RAF family molecules, avoiding paradoxical activation of the MAPK pathway as well. Belvarafenib—an oral type II pan-RAF kinase inhibitor—exhibited promising antitumor activity in a phase I trial enrolling patients with advanced solid tumors with RAS or RAF mutations [101]. Efficacy and safety of the RAF dimer selective inhibitor naporafenib, in combination with trametinib or with the ERK inhibitor LTT462, is being explored in another phase I clinical trial (NCT02974725) in advanced or metastatic KRAS- or BRAF-mutant NSCLC progressed following standard therapy. Plixorafenib (FORE8394), another selective dimer inhibitor of BRAF, combined with binimetinib, showed robust anticancer activity in cells harboring BRAF V600 or non-V600 mutations or BRAF fusions, higher than the other combinations tested (binimetinib plus vemurafenib or tovorafenib or lifirafenib), representing a promising new strategy in patients with acquired resistance [102]. PF-07799933 is another new BRAF dimers inhibitor that showed in vitro efficacy in BRAF V600E mutated cells (with even a greater in vitro potency than plixorafenib) and is currently being evaluated in a phase I study (NCT05355701).

Finally, the preliminary efficacy of ABM-1310—another selective BRAF inhibitor—either alone or in combination with cobimetinib, was seen in 51 patients with advanced BRAF V600-mutated solid tumors, including those who failed previous BRAF ± MEK inhibitor treatment (n = 38) [103]. Specific data regarding NSCLC are missing.

As regards the co-targeting of MEK, a new generation of selective inhibitors is under evaluation. For example, IMM-6-415, a third-generation dual MEK inhibitor, is being evaluated in a phase I study involving RAS and RAF mutated solid tumors. ABM-168 is another MEK inhibitor that showed in vitro high activity in multiple cancer cell lines with BRAF, RAS, or NF1 mutations and is currently under evaluation in a phase I first-in-human clinical trial [104].

All these results support the potential role of next-generation RAF inhibitors beyond BRAF-mutant NSCLC, but further exploration is urgently needed. Moreover, due to the evolving landscape of precision medicine and targeted therapies, they highlight the central role of a comprehensive molecular characterization, both at diagnosis and the time of progression, to detect co-occurring mutations and possible acquired resistance mechanisms, guiding the selection of the optimal treatment strategy.

Of note, the implementation of a prolonged on-off treatment schedule or the introduction of “drug holidays” has been suggested as an alternative strategy in melanoma to promote the expansion of sensitive clones to targeted therapy while allowing for the reduction of resistant clones, thereby preserving tumor responsiveness. However, the real benefit of this approach is uncertain [105].

## 5. Conclusions

Despite the rarity of BRAF mutations in NSCLC, research in this area must continue, as many unresolved questions remain. A better understanding of the molecular mechanisms underlying the BRAF pathway and the tumor microenvironment associated with these mutations is essential for accurately characterizing and targeting non-V600 mutations. This knowledge will also aid in integrating targeted therapies with immunotherapy as it appears to be effective in these patients; in particular, ICIs could also be considered as monotherapy in those with BRAF non-V600 mutations, high expression of PD-L1 or TMB and a history of smoking. In addition, exploring new combinations strategies or new molecules (ERK1/2 inhibitors, next-generation RAF inhibitors) to overcome resistance mechanisms is crucial to providing personalized treatment and an effective therapeutic sequence for our patients. This further highlights the relevance of performing tumor tissue rebiopsy at the time of progression and the importance of early access to clinical trials.

## Figures and Tables

**Table 1:** Most frequent BRAF mutations in NSCLC.

Mutation	Class	Nucleotide Change	AA Change	Location in the Protein	Effect
V600E	I	1799	T>A	Activation segment	Activate kinase activity
V600K	I	1798_1799	delinsAA	Activation segment	Activate kinase activity
V600R	I	1798_1799	GT>AG	Activation segment	Activate kinase activity
L597Q	II	1790	T>A	Activation segment	Activate kinase activity
L597R	II	1790	T>G	Activation segment	Activate kinase activity
K6001E	II	1801	A>G	Activation segment	Activate kinase activity
G464V	II	1391	G>T	P-loop	Activate kinase activity
G469A	II	1406	G>C	Glycine-rich region	Activate kinase activity
G469E	II	1406	G>A	Glycine-rich region	Unknown
G469R	II	1405	G>A	Glycine-rich region	Unknown
G466R	III	1396	G>A	P-loop	Impaired kinase activity
G466A	III	1397	G>C	P-loop	Activate kinase activity
G466V	III	1397	G>T	P-loop	Impaired kinase activity
D594E	III	1782	T>G	DFG motif	Impaired kinase activity
D594G	III	1781	A>G	DFG motif	Impaired kinase activity
D594H	III	1780	G>C	DFG motif	Impaired kinase activity
D594N	III	1780	G>A	DFG motif	Impaired kinase activity
N581Sa	III	1742	A>G	Catalytic loop	Activate kinase activity
G596R	III	1786	G>C	DFG motif	Impaired kinase activity

**Table 2:** Case reports of BRAF non-V600 NSCLC.

BRAF Mutation	Co-Mutations	Detection Technique	Drug	Line	Best Response	PFS	Case Report
**G469L**	NR	Sanger sequencing	Vemurafenib	1	PD	2 weeks	Gautschi et al. [27]
**G469A**	TP53	NGS	Dabrafenib/trametinib	4	SD	6 months	Dagogo-Jack et al. [28]
**G469V**	*APC* R1040fs∗16 and CHD2 L1383∗ mutations and *NFKBIA* and *NKX2-1* amplifications	NGS	Dabrafenib/trametinib	2		9 weeks	Negrao et al. [18]
**L597R**	none	NGS	Dabrafenib/trametinib	2	PR	NR at 12 months	Negrao et al. [18]
**D594G**	*TP53* H193L	NGS	trametinib	4	SD	4 months	Negrao et al. [18]
**G469A and W604C**	*none*	NGS	Dabrafenib/trametinib	1	PR	NR at 15 months	Reyes et al. [29]
**G469R**	*TP53 E68, ATRE2209, STK11S69, ATK11 F354L mutations; PI3KCA, PDGFRA, Kit, KDR amplifications and CDKN2A loss.*	NGS	Sorafenib	8	PR	6 months	Sereno et al. [30]
**p.T599dup**	*none*	sanger sequencing and NGS	Dabrafenib/trametinib	1	PR	NR at 4 months	Turshudzhyan et al. [31]
**p.T599dup**	*TP53 exon 8 p.V272M missense mutation, BRCA2 exon 11 missense mutation andCTNNB1 exon 3 missense mutation.*	NGS	Dabrafenib/trametinib	1	SD	8 months	Jiang et al. [32]
**G596R**	*NR*	NGS	CT-IO	1	PR	6 months	Lazar et al. [33]
**G464V**	*NR*	NGS	CT	1	PD	2 weeks	Lazar et al. [33]
**G469V**	*NR*	NGS	CT-IO	1	PR	NR at 18 weeks	Lazar et al. [33]
**G469A**	*NR*	NGS	IO	2	PR	NR at 4 years	Rittberg et al. [34]
**E501Q**	*none*	NGS	CT-IO	1	PR	NR at 3 years	Do et al. [35]
**K601E**	*EGFR amplification (copy number: 2.36) and RICTOR amplification (copy number: 2.74)*	NGS	Dabrafenib/trametinib	1	PR	9 months	Su et al. [36]
**G469V**	*none*	PCR	Sorafenib	2	PR	13 months	Casadei-Gardini et al. [37] Concomitant HCC
**K601E**	*FGFR4 and ALK VUS*	NGS	trametinib	3	PR	4 months	Saalfeld et al. [38]
**G466R**	NF1 I70fs*15	NGS	Dabrafenib/trametinib	3	PD	3 months	Citarella et al. [39]

PFS = progression-free survival, PD = progression disease, SD = stable disease, PR = partial response, CR = complete response, NGS = next-generation sequencing, NR = not reached.

**Table 3:** Ongoing trials in BRAF-mutated NSCLC. BRAFi = BRAF inhibitor, NOS = not otherwise specified, RAFi = RAF inhibitor, MEKi = MEK inhibitor, ICI = immune checkpoint inhibitor, NS = not specified, NSCLC = non-small cell lung cancer, ULK1/2i = ULK1/2 inhibitor.

Trial	Phase	Mutation	Setting	Experimental Arm
NCT06054191	II	BRAF V600	perioperatory	Dabrafenib plus trametinib
NCT05800340	II	BRAF (any confirmed driver)	Neoadjuvant	Toripalimab plus chemotherapy
NCT06563999	II	BRAF V600E	Neoadjuvant	Dabrafenib plus trametinib
NCT05786924	I	All BRAF and CRAF	2nd line	BDTX-4933 (BRAFi)
NCT05355701	I	All BRAF	Metastatic NOS	PF-07799933 (BRAFi) +/− binimetinib
NCT06270082	I	BRAF class II-III and fusions	Metastatic NOS	IK-595 (Dual MEK/RAFi)
NCT04913285	Ib	All BRAF	No previous BRAFi allowed	KIN-2787 (RAFi) +/− binimetinib
NCT06326411	I	MAPK pathway	No previous BRAFi allowed	NST-628 (Pan-RAF/MEKi)
NCT05501912	I	BRAF V600	Advanced	ABM-1310 (BRAFi)
NCT05538130	I	BRAF class II/III	Metastatic NOS	PF-07799544 (MEKi) +/− PF-07799933
NCT03284502	I	All RAF	Metastatic NOS	HM95573 (RAFi) + cobimetinib
NCT02407509	I	RAS-RAF-MEK	Metastatic NOS	VS-6766 (RAF/MEki) +/− everolimus
NCT04965220	I	All BRAF	No previous BRAFi allowed	HLX208 (BRAF V600E Inhibitor)
NCT05275374	I-IIa	BRAF V600	Advanced	XP-102 (RAFi) plus trametinib
NCT05641493	Ib-II	BRAF V600E	No previous BRAFi or ICI allowed	HLX208 plus serplulimab (anti-PD-1 monoclonal antibody)
NCT05900219	II	BRAF V600E	Advanced	HL-085 (MEKi) plus Vemurafenib
NCT05580770	I-IIa	MAPK pathway, NSCLC cohort BRAF class II-III	Metastatic NOS	BGB-3245 (RAFi)
NCT06287463	I-II	MAPK pathway	NS (prior treatment with certain BRAF dimer inhibitors not allowed)	DCC-3084 (RAFi)
NCT04985604	Ib-II	MAPK pathway	Metastatic NOS	Tovorafenib (RAFi)
NCT04892017	I-II	RAS, NF1, or RAF	2nd line and beyond	DCC-3116 (ULK1/2i)
NCT06074588	III	BRAF V600E	3rd line and beyond	Sacituzumab Tirumotecan (MK-2870) vs. docetaxel/pemetrexed

## Data Availability

Data sharing is not applicable to this article as no new data were created or analyzed in this study.
